# Prevalence of symptomatic dry eye and influencing factors among Chinese adolescents: A cross-sectional study

**DOI:** 10.1371/journal.pone.0312725

**Published:** 2024-10-29

**Authors:** Xiaojuan Chen, Yue Zhou, Xian Gao, Yan Zhu, Qi Cai, Bianyu Yin, ZhiMin Sun, Yaojia Xiong, Yong Wang, Xiaobo Huang

**Affiliations:** 1 Department of Ophthalmology, Second Affiliated Hospital of Nantong University, Nantong, Jiangsu Province, China; 2 Department of Endocrinology, Second Affiliated Hospital of Nantong University, Nantong, Jiangsu Province, China; Peking University Third Hospital, CHINA

## Abstract

**Background:**

Comprehensive research on the impact of various types of refractive errors (RE) and anisometropia on dry eye disease is still lacking. This study aimed to estimate the prevalence rates and potential lifestyle factors related to symptomatic dry eye (SDE) among adolescents in eastern China.

**Methods:**

A cross-sectional study was performed in 2023, and a stratified cluster sampling technique was used among adolescents in Nantong, China. Demographic information, including sex, age and BMI, were collected. All participants underwent optometric tests, while Ocular Surface Disease Index (OSDI) and self-designed questionnaires were administered. Both univariate and multivariate logistic regression analyses were used to assess associations between SDE and related parameters, and various types of RE and anisometropia were also included in the study.

**Results:**

A total of 1,518 participants were enrolled in the study, and the overall prevalence of SDE was 20.3% among adolescents in Nantong, China. Multiple logistic regression analyses showed that high myopia (aOR = 3.42, 95% CI = 1.60–3.36, p = 0.025), frequent use of eye drops (aOR = 2.31, 95% CI = 1.60–3.36, p<0.001), a history of allergic conjunctivitis (aOR = 1.93, 95% CI = 1.09–3.34, p = 0.025), and frequent blinking (aOR = 3.23, 95% CI = 2.31–4.53, p<0.001) were identified as risk factors for SDE. Conversely, male gender (aOR: 0.76, 95% CI: 0.58–0.99, p = 0.043), increased sleep time (6–7 h: aOR = 0.64, 95% CI = 0.46–0.89, p = 0.009; 7–8 h: aOR = 0.64, 95% CI = 0.43–0.95, p = 0.026; >8 h: aOR = 0.43, 95% CI = 0.23–0.82, p = 0.010), and timely intervention when vision decline occurred were protective factors against SDE (aOR = 0.61, 95% CI = 0.43–0.85, p = 0.004).

**Conclusion:**

High myopia was found to be independently associated with an increased risk of SDE., while hyperopia, astigmatism, and anisometropia were not independent risk factors for SDE. The identified risk and protective factors may help provide valuable insights for future research and interventions aimed at improving ocular health in adolescents.

## Introduction

According to the definition given in the International Dry Eye Work Shop (DEWS) report, dry eye disease is a multifactorial disease of the ocular surface characterized by a loss of tear film homeostasis and accompanied by ocular symptoms [1]. Epidemiological investigation indicated that the prevalence of symptomatic dry eye (SDE) ranged from 5% to 50% cross various regions and populations [1, 2], and the symptoms include ocular pain, redness, itchiness, burning, irritation, photophobia, dryness, foreign body sensation, and visual symptoms, notably fluctuating or blurred vision [3], which can interfere with daily activities and affect vision-related quality of life [4, 5]. The vast majority of current studies on SDE have focused on adults aged 20–96 years rather than adolescents [6–8]. Given the high prevalence of SDE among adolescents and the limited research in this area, further exploration is essential.

Refractive errors (RE) are intrinsic eye disorders associated with several ocular problems, consist of three main types: myopia, hyperopia and astigmatism [9]. RE can result in defects within components of the ocular system due to restraining light from focusing on the retina. Previous studies on RE and SDE mainly concentrated on the correlation between myopia and SDE [10–12]. A few studies also analyzed the relationship among myopia, hyperopia and SDE simultaneously [13], but the relevance of astigmatism, anisometropia and SDE remains unclear. Thus, the relationship between different types of RE and SDE, especially in adolescents, needs further exploration. Given the current high prevalence of RE in the adolescent population [14, 15], elucidating the relationship between different types of RE and SDE in adolescents may help avoid more visual complications, improve quality of life, and increase educational opportunities for more adolescents.

In addition, multiple risk factors for SDE in adults have been illustrated. For example, the sign and symptoms of allergic conjunctivitis often overlap with SDE, and can directly cause tear film instability, thus aggravating SDE. Frequent use of eye drops impairs blinking patterns, disturbs meibum distribution and decreases the exposure of the eye surface to tear film, which also triggers SDE [16–18]. However, relevant research on risk factors of SDE for adolescents is still inadequate. On the one hand, the descriptions of SDE in adolescents tend to be unclear, making accurate diagnosis difficult, and can even be ignored by physicians. On the other hand, the existing few studies indicate that SDE can exert negative effects on adolescents’ academic performance, general well-being, long-term eye health and so on [19, 20]. Rapid transformation of living environment accompanies with the changes of adolescents’ lifestyles and the increasing prevalence of ocular surface diseases in adolescents, thereby underscoring the escalating importance of investigating the association between lifestyle factors and SDE among adolescents.

Quantifying ocular symptoms through questionnaires is a key screening tool to determine if further tests are needed [21]. The Ocular Surface Disease Index (OSDI) has been proven to be a highly reliable test and is probably the most widely used questionnaire for the clinical research and screening of SDE at present to reduce survey bias [22]. Herein, this study aims to evaluate the prevalence of SDE in middle school students in Nantong through questionnaires and explore the relationship between different types of RE and various potential lifestyle factors and SDE, thus providing theoretical basis for the development of corresponding intervention measures in the future.

## Methods

### Study design

The present study is part of the Nantong School-aged Children Eye Study (NSES), which has been registered in the Chinese Clinical Trial Registry (http://www.chictr.org. cn, ChiCTR2300077367). The NSES is a school-based study of ocular conditions in Nantong, a medium-sized prefecture level city in eastern China with a relatively stable population profile. The current study evaluates the relationship between RE, lifestyle factors and SDE. In September 2023, a cross-sectional study was conducted. The study was approved by the ethics committee of the Second Affiliated Hospital of Nantong University, China (approval number: 2023KT122). All protocols used in this study followed the tenets of the Declaration of Helsinki [23]. Participants under the age of 18 obtained written informed consent from at least one guardian, and participants aged 18 or older should directly obtain written informed consent.

### Sample selection

The prevalence of SDE in Nantong in 2021 from our previous study was evaluated at 19.55% [24]. To achieve a power of 80%, the sample size was calculated using the formula (n = t^2^pq/d^2^) [14], and assumed a design effect of 1.5 and a nonresponse rate of 5% via cluster sampling (t = 2 for a 95% confidence interval (CI), q = 1-P, d = 0.1 P). The total sample size was at least 1,646.

A stratified cluster sampling method was performed. The cluster was stratified by grade and age. Classes in each grade were selected by simple random sampling, and all students in these classes were required to participate in the study. Participants who uncooperated with the examination, were unable to complete the questionnaires independently or with the accompaniment of a guardian, had a history of eye surgery, missing data, incorrect information, and the best corrected visual acuity (BCVA)<0.8 were excluded from the analysis. Junior high school and senior high school students from 14 schools (7 junior high schools and 7 senior high schools) in Nantong were included. One class was randomly selected from each grade of each school, where the inspection site is set. The proportion of adolescents who volunteered to participate in the invitation was ultimately 95%.

To standardize the lighting and test distance, researchers visited and arranged each venue carefully before the study began. Autorefractors were calibrated every day. All optometric tests were performed by three trained ophthalmologists. Non-cycloplegic autorefractive examination was conducted with a microscope (WSRMK-8000, bibase, Shandong, China) and repeated three times. If the difference between any two of the three results exceeded 0.50 D (diopter), additional tests would be executed immediately. The average of the three appropriate results was then calculated. Uncorrected visual acuity and BCVA were measured at 5 m via a standard logarithmic liquid crystal roll E chart (WSVC-100, Qingda Optometry, Berkeley, CA, USA). Calibration of BCVA based on autorefraction results, and was recorded in the student’s online refraction file.

### Questionnaires

All participants and their parents completed a detailed questionnaire with the help of a well-trained investigator. The questionnaire for the present study was mainly composed of two different parts. The first part was the Chinese version of the modified OSDI, which has been acknowledged as the most common SDE-specific questionnaire to assess subjective ocular symptoms. The questionnaire has been validated in Chinese language and contained 11 questions to measure the frequency of ocular symptoms, vision-related functions and limitation, and environmental triggers in the previous week [25]. The question about night driving was omitted as adolescents are not expected to drive during nighttime. Each response was scored using a 5-point scale whereby 0 indicates no problem and 4 indicates a significant problem. The total OSDI score was calculated as follows: (sum of scores×25/11) and ranged from 0 to 100 [1].

The second part was the self-designed questionnaire, which could be available online (S1 File). The questionnaire was developed through comprehensive consideration in the TFOS lifestyle report [26], previous research [27], and the actual situation of Chinese teenagers. It consisted of the basic information of participants, such as sex, age, and BMI, and factors which might cause SDE, including frequent eye drops use, contact lens wear, spectacles use, eating habits, academic burden, daily TV watching time, daily mobile phone or iPad use time, daily homework time, daily sleep time, daily outdoor activity, timely intervention when vision decline occurred, history of allergic conjunctivitis, history of chalazion, frequent blinking and so on.

Frequent use of eye drops was defined as using eye drops at least once a week in the previous 3 months [8]. Contact lens wear was defined as using contact lenses at least once a week in the past 3 months [8]. The mean number of hours spent outdoors each day was calculated using the following formula [28]: [(hours spent on a weekday)×5+(hours spent on a weekend day)×2 divided by 7]. A history of allergic conjunctivitis refers to eye itching within the past 3 months, diagnosed by an ophthalmologist as allergic conjunctivitis [29]. Frequent blinking was defined as >20 blinks/min or abnormal blinks, such as winking and frowning [30].

### Definitions

In the current study, students with an OSDI score of ≥13 are considered to have "SDE" [31]. Referring to the refractive error study in children surveys [32], spherical equivalent refraction (SE) was calculated as follows: SE = negative cylindrical degree×0.5+spherical degree. Myopia was defined as SE≤-0.5 D. Low myopia was defined as -3.0 D<SE≤-0.5D. Moderate myopia was defined as -6.0 D<SE≤-3.0 D. High myopia was defined as an SE≤-6.0 D. Emmetropia was defined as -0.5 D<SE≤0.5 D. Hyperopia was defined as an SE>+0.5 D. Anisometropia was defined as the absolute SE difference ≥1.0 D between eyes. Astigmatism was defined as cylinder power 1.0 D or greater [33, 34]. According to the axis position, with-the-rule (WTR) was negative cylinder axis falling between 1° and 30° or 150° and 180°, against-the-rule (ATR) was a negative cylinder axis falling between 60° and 120°, and oblique (OBL) in other orientations.

### Statistical analysis

SPSS statistical software, Windows version 22 (SPSS, Chicago, Illinois, USA), was used for data processing. For the description of patients’ characteristics, descriptive statistics were calculated; mean±standard deviation (SD) was used for continuous variables, while relative frequencies and percentages for categorical variables were reported. The prevalence rate of SDE was described in simple proportion. The polynomial linear correlation in one-way ANOVA was used for trend test (Ptrend). Group comparisons were done by the Chi-squared (χ^2^) test or Fisher’s exact test for categorical variables. SDE was considered as the dependent variable. Factors that showed a univariate association with a value of P<0.1 were selected as candidate variates for multivariate analyses. In multivariate logistic regression, all variables for multivariate logistic regression analysis were examined for multicollinearity, the variance inflation factors for all variables were less than 5. The multivariable logistic regression model was established by forward stepwise selection. The Hosmer-Lemeshow test showed P>0.05 (x^2^ = 5.59, P = 0.693). The odds ratio (OR) and 95% CI for the associated factors were calculated and expressed as adjusted OR (aOR) in the multivariate logistic regression analysis. A value of P<0.05 was considered statistically significant.

## Results

A total of 1,680 participants were invited to participate in the study. The completion percentage of participants within junior and senior high school students in Nantong was 0.63%. A total of 1,518 participants were included in the statistical analysis, with 759 (50.0%) females. The response rate of participants was 90.4%. The mean age was 14.68±1.70 years, ranging from 12–18 years. As illustrated in Table 1, the overall prevalence of SDE, myopia, hyperopia, astigmatism and anisometropia were 20.3%, 92.6%, 0.9%, 41.0%, 32.8%, respectively, and did not show a trend of change with age (all, Ptrend>0.05).

**Table 1:** Prevalence of SDE and refractive error stratified by age.

Age	n	Prevalence of SDE	Prevalence of myopia	Prevalence of hyperopia	Prevalence of astigmatism	Prevalence of anisometropia
12	157 (10.3%)	21.7%	91.7%	0.0%	38.2%	30.6%
13	292 (19.2%)	21.6%	93.8%	0.7%	39.7%	34.9%
14	277 (18.2%)	19.9%	93.5%	1.1%	44.4%	32.5%
15	283 (18.6%)	20.1%	90.8%	2.1%	47.3%	31.1%
16	263 (17.3%)	18.3%	93.2%	0.8%	38.0%	30.8%
17	160 (10.5%)	23.8%	90.6%	0.6%	38.8%	33.1%
18	86 (5.7%)	15.1%	95.3%	0.0%	32.6%	41.9%
Total	1518 (100%)	20.3%	92.6%	0.9%	41.0%	32.8%
χ 2 (F)		0.844	0.119	0.010	1.275	1.775
P-value		0.358	0.730	0.919	0.259	0.183

Results of Chi-square test for trend test (Ptrend). SDE, symptomatic dry eye.

Table 2 list the results of univariate and multiple logistic regression analyses. Univariate logistic regression analysis indicated that participants with male gender was a protective factor for SDE (OR: 0.72, 95% CI: 0.56–0.93, p = 0.011). In terms of the possibility for suffering from SDE, participants with high myopia were 4.26 times more likely than those without myopia (OR: 4.26, 95% CI: 1.48–12.24, p = 0.007); participants who use eye drops more than three times a week were 2.63 times more likely than those without eye drops (OR: 2.63, 95% CI: 1.86–3.72, p<0.001); participants with daily homework time more than 4 h were 2.10 times more likely than those less than 1 h (OR: 2.10, 95% CI: 1.02–4.30, p = 0.043); participants with history of allergic conjunctivitis were 2.54 times more likely than those without (OR: 2.54, 95% CI: 1.50–4.29, p = 0.001); participants who frequent blinking were 3.59 times more likely than those without (OR: 3.59, 95% CI: 2.60–4.97, p<0.001). Participants who were noticed a decline in vision would be taken to a medical institution for examination by their parents, and this was a protective factor for SDE (OR: 0.61, 95% CI: 0.44–0.83, p = 0.002). Additionally, the prevalence of SDE in participants who slept <6 h a day was 28.5%. In comparison, participants whose sleep times were 6–7 h, 7–8 h and >8 h per day had significantly lower prevalence of SDE (OR: 0.59, 95% CI: 0.43–0.81, p = 0.001; OR: 0.57, 95% CI: 0.39–0.82, p = 0.003; OR: 0.49, 95% CI: 0.24–0.83, p = 0.010, respectively).

**Table 2:** Analysis of associated factors for SDE in Chinese adolescents.

Variables	Levels	Non-SDE (N = 1,210)	SDE (N = 308)	OR (univariate analysis)	OR (multivariate analysis)
Sex, n (%)	Female	625(%)	134(%)		
	Male	585(%)	174(%)	0.72 (0.56–0.93, p = 0.011)	0.76 (0.58–0.99, p = 0.043)
Age (years)		14.69±1.70	14.61±1.70	0.97 (0.90–1.05, p = 0.444)	
BMI		21.10±3.82	20.68±3.71	0.97 (0.94–1.00, p = 0.084)	
Myopia, n (%)	Non myopia	43 (3.6%)	4 (1.3%)		
	Low myopia	407 (33.6%)	89 (28.9%)	2.35 (0.82–6.71, p = 0.111)	1.95 (0.67–5.68, p = 0.223)
	Moderate myopia	548 (45.3%)	131 (42.5%)	2.57 (0.91–7.28 p = 0.076)	2.03 (0.70–5.86, p = 0.193)
	High myopia	212 (17.5%)	84 (27.3%)	4.26 (1.48–12.24, p = 0.007)	3.42 (1.60–3.36, p = 0.025)
Hyperopia, n (%)	No	1,197 (98.9%)	307 (99.7%)		
	Yes	13 (1.1%)	1 (0.3%)	0.3 (0.03–2.30, p = 0.247)	
Astigmatism, n (%)	No	713 (58.9%)	182 (59.1%)		
	Yes	497 (41.1%)	126 (40.9%)	0.99 (0.77–1.28, p = 0.958)	
Anisometropia, n (%)	No	814 (67.3%)	206 (66.9%)		
	Yes	396 (32.7%)	102 (33.1%)	1.02 (0.78–1.33, p = 0.897)	
Use eye drops more than three times a week	No	1,108 (91.6%)	248 (80.5%)		
	Yes	102 (8.4%)	60 (19.5%)	2.63 (1.86–3.72, p<0.001)	2.31 (1.60–3.36, p<0.001)
Contact lens wear	No	1168 (96.5%)	291 (94.5%)		
	Yes	42 (3.5%)	17 (5.5%)	1.63 (0.91–2.90, p = 0.100)	
Use of spectacles	No	337 (27.9%)	72 (23.4%)		
	Yes	873 (72.1%)	236 (76.6%)	1.27 (0.94–1.70, p = 0.115)	
Have good eating habits	No	545 (45.0%)	121 (39.3%)		
	Yes	665 (55.0%)	187 (60.7%)	1.27 (0.98–1.64, p = 0.070)	
Academic burden	Normal	794 (65.6%)	189 (61.4%)		
	Serious	416 (34.4%)	119 (38.6%)	1.27 (0.98–1.64, p = 0.163)	
Daily TV watching time	<0.5 h	856 (70.7%)	210 (68.2%)		
	0.5–1 h	188 (15.5%)	51 (16.6%)	1.11 (0.78–1.56, p = 0.567)	
	1–1.5 h	97 (8.0%)	24 (7.8%)	1.01 (0.63–1.62, p = 0.972)	
	1.5–2 h	32 (2.6%)	9 (2.9%)	1.15 (0.54–2.44, p = 0.723)	
	>2 h	37 (3.1%)	14 (4.5%)	1.54 (0.82–2.91, p = 0.180)	
Daily mobile phone or iPad use time	<0.5 h	295 (24.4%)	63 (20.5%)		
	0.5–1 h	262 (21.7%)	67 (21.8%)	1.20 (0.82–1.76, p = 0.355)	
	1–1.5 h	283 (23.4%)	67 (21.8%)	1.11 (0.76–1.62, p = 0.596)	
	1.5–2 h	206 (17.0%)	61 (19.8%)	1.39 (0.94–2.06, p = 0.104)	
	>2 h	164 (13.6%)	50 (16.2%)	1.43 (0.94–2.17, p = 0.095)	
Daily homework time	<1 h	57 (4.7%)	10 (3.2%)		
	1–2 h	299 (24.7%)	56 (18.2%)	1.07 (0.51–2.22, p = 0.861)	
	2–3 h	370 (30.6%)	82 (26.6%)	1.26 (0.62–2.58, p = 0.521)	
	3–4 h	261 (21.6%)	78 (25.3%)	1.70 (0.83–3.49, p = 0.146)	
	>4 h	223 (18.4%)	82 (26.6%)	2.10 (1.02–4.30, p = 0.043)	
Daily sleep time	<6 h	188 (15.5%)	75 (24.4%)		
	6–7 h	641 (53.0%)	151 (49.0%)	0.59 (0.43–0.81, p = 0.001)	0.64(0.46–0.89, p = 0.009)
	7–8 h	297 (24.5%)	67 (21.8%)	0.57 (0.39–0.82, p = 0.003)	0.64(0.43–0.95, p = 0.026)
	>8 h	84 (6.9%)	15 (4.9%)	0.49 (0.24–0.83, p = 0.010)	0.43(0.23–0.82, p = 0.010)
Daily outdoor activity	<0.5 h	405 (33.5%)	126 (40.9%)		
	0.5–1 h	527 (43.6%)	113 (36.7%)	0.69 (0.52–0.92, p = 0.010)	
	1–2 h	164 (13.6%)	43 (14.0%)	0.84 (0.57–1.25, p = 0.391)	
	2–3 h	59 (4.9%)	10 (3.2%)	0.55 (0.27–1.10, p = 0.089)	
	>3 h	55 (4.5%)	16 (5.2%)	0.94 (0.52–1.69, p = 0.824)	
Timely intervention when vision decline occurred	No	169 (14.0%)	65 (21.1%)		
	Yes	1,041 (86.0%)	243 (78.9%)	0.61 (0.44–0.83, p = 0.002)	0.61 (0.43–0.85, p = 0.004)
History of allergic conjunctivitis	No	1,171 (96.8%)	284 (92.2%)		
	Yes	39 (3.2%)	24 (7.8%)	2.54 (1.50–4.29, p = 0.001)	1.93 (1.09–3.34, p = 0.025)
History of chalazion	No	1,112 (91.9%)	280 (90.9%)		
	Yes	98 (8.1%)	28 (9.1%)	1.14 (0.73–1.76, p = 0.573)	
Frequent blinking	No	1,104 (91.2%)	229 (74.4%)		
	Yes	106 (8.8%)	79 (25.6%)	3.59 (2.60–4.97, p<0.001)	3.23 (2.31–4.53, p<0.001)
Where mainly stay during class intervals	Classroom	399 (33.0%)	94 (30.5%)		
	Outdoor	811 (67.0%)	214 (69.5%)	1.12 (0.86–1.47, p = 0.411)	

SDE, symptomatic dry eye; Timely intervention when vision decline occurred, parents taking participants to medical institutions for examination when vision decline occurred.

After adjustment for other characteristics, the results still showed that participants with male gender was a protective factor for SDE (aOR: 0.76, 95% CI: 0.58–0.99, p = 0.043). For the possibility of developing SDE, participants with high myopia were 3.42 times more likely than those without myopia (aOR: 3.42, 95% CI: 1.60–3.36, p = 0.025); participants who use eye drops more than three times a week were 2.63 times more likely than those without eye drops (aOR: 2.31, 95% CI: 1.60–3.36, p<0.001); participants with history of allergic conjunctivitis were 1.93 times more likely than those without (aOR: 1.93, 95% CI: 1.09–3.34, p = 0.025); participants who frequent blinking were 3.23 times more likely than those without (aOR: 3.23, 95% CI: 2.31–4.53, p<0.001). Participants who were noticed a decline in vision would be taken to a medical institution for examination by their parents, and this was a protective factor for SDE (aOR: 0.61, 95% CI: 0.43–0.85, p = 0.004). In comparison with those <6 h a day, participants whose sleep times were 6–7 h, 7–8 h and >8 h per day had significantly lower prevalence of SDE (aOR: 0.64, 95% CI: 0.46–0.89, p = 0.009; aOR: 0.64, 95% CI: 0.43–0.95, p = 0.026; aOR: 0.43, 95% CI: 0.23–0.82, p = 0.010, respectively).

### Discussion

The current research aimed to identify the prevalence and related factors of SDE in Chinese adolescents. Since previous studies only involved the effect of myopia on SDE [10, 12], this study comprehensively considered the relationship among various types of RE, anisometropia, lifestyle factors and SDE, thus potentially filling the gap in this research field.

The prevalence of SDE globally is 5% to 50% [35]. The onset of SDE has recently been found to become prevalent in younger age groups, with an increasing prevalence in children and adolescents [36]. The prevalence of SDE in Chinese children has confirmed to be relatively high. According to the epidemiological survey of children aged 7–8 years in Nanjing, Jiangsu Province in 2019, the prevalence of SDE in children was 8.7% [37]. The epidemiological survey of high school students in Shandong Province in 2010 revealed that the prevalence of SDE in Chinese adolescents was 23.7% [8]. The epidemiological survey of children aged 10–14 years in Cangzhou City, Hebei Province in 2011 revealed a SDE prevalence of 28.53% [38]. Our results also indicate a high prevalence of SDE among adolescents (20.3%).

To date, few studies have comprehensively evaluated the relationship between various types of RE and SDE, especially in adolescents. Factors such as myopia, hyperopia, astigmatism, and anisometropia were considered in the current study. Due to the high prevalence of myopia (92.6%), further investigation was conducted on different degrees of myopia. The results showed that high myopia was a risk factor for SDE, while hyperopia, astigmatism, and anisometropia were not. Simultaneously, according to the multiple regression analysis, the risk of SDE raised with the increase of degree of myopia compared with non-myopic participants. In line with our study, adult participants with high myopia have been found a higher prevalence of SDE [12]. On the one hand, the increased orbital volume caused by the axial elongation for participants with high myopia may result in proptosis [39], thus inducing SDE due to exposure keratopathy [40]. On the other hand, Hazra et al. [41] suggest that the association between SDE and myopia may involve the parasympathetic nervous system, as it affects the lacrimal gland and the thickness of choroid, which may be an upstream factor for the association above.

In addition to RE and anisometropia, other factors related to visual conditions, such as low vision [42] or insufficient refractive correction [8, 37], have also been shown to be potentially associated with SDE. In the low vision rehabilitation service at the University of Colorado, SDE in low vision populations is an important comorbidity that occurs in more than one-third of patients. As the study on DED in Chinese children shown [37], 2.04% students had insufficient refractive correction and were associated with "definite DED", indicating that insufficient refractive correction might be one of the causes of ocular surface damage in Chinese students. Our results supported this hypothesis. In the current study, timely intervention when vision decline occurred was an important protective factor for SDE. Adolescents in the study population received at least one vision examination organized by the education department every year. Transitioning from school examinations to further medical institution treatment may present problems. Insufficient access to healthcare is a crucial reason for the lack of follow-up healthcare [43].

Allergic conjunctivitis was identified as an important risk factor for SDE in our study. Participants with allergic conjunctivitis had a 1.93-fold higher prevalence of SDE compared to participants without allergic conjunctivitis. SDE and allergic conjunctivitis are well-known as the two most common ocular surface diseases [44] negatively affecting one’s quality of life and work productivity [45]. The underlying pathophysiology of SDE and allergic conjunctivitis has been reported to root in immunological changes that lead to ocular surface inflammation, and their common pathogenesis paves the way for exacerbating the negative synergistic effects of other diseases [46, 47]. Given that SDE and allergic conjunctivitis share common ocular symptoms and risk factors, it is important to appropriately diagnose and treat these diseases and their complications. The prevalence of allergic conjunctivitis among adolescents in Brazil, as indicated by the International Study of Asthma and Allergies in Childhood Core Questionnaire, was estimated to be 20.7% [48]. In a similar study in Shanghai, the prevalence of allergic conjunctivitis among adolescents aged 12–14 years was approximately 27% [29]. Unfortunately, the current study did not conduct a core questionnaire survey on allergic conjunctivitis.

Our results indicated that participants who frequently used eye drops had a 2.31-fold higher prevalence of SDE, consist with the research from Shandong, China [8]. Since there are no accurate statistics on the use and type of eye drops, the relationship between SDE and eye drop use needs to be interpreted with caution. On the one hand, 10.7% participants have been found to self-use eye drops, due to the fact that most eye drops in China can be purchased without a prescription, thus resulting in the abuse of eye drops among adolescents (such as drugs related to myopia prevention and control). Local use of eye drops may have immune inflammatory effects on the cornea, conjunctiva, meibomian glands, and corneal nerves [49, 50], and frequent use of eye drops containing preservatives may lead to SDE [51]. On the other hand, since the limitations of cross-sectional studies, it was impossible to determine the causal relationship between SDE and eye drop use directly in the current study.

Although contact lens wear has been verified as a persistent risk factor for SDE in adults [2], the situation in children and adolescents is still unclear. In a few early studies based on high school students, contact lens wear was shown to be an independent risk factor for SDE [8, 52]. Interestingly, accumulated studies have recently tended to suggest that contact lens wear is not an independent risk factor for SDE [37, 53]. The current result on contact lens wear was in accordance with the findings above, displaying no association with SDE in adolescent. This might be due to the fact that adolescents who wear contact lenses have fewer SDE than adults. What’s more, it is closely connected with the increasing standardization of contact lens wear and management, thus further controlling serious adverse reactions timely and effectively [54].

Additionally, several risk and protective factors have been identified. For example, frequent blinking had a significant correlation with SDE, which could be attributed to the instability of the tear film, thus exacerbating by dry eye and rendering the motor response hypersensitive. Alternatively, the instability of the tear film might also appear in conditions such as benign essential blepharospasm [55]. Similar to most previous studies, female participants were found to be at a higher risk of SDE [2, 52, 56, 57], and short sleep duration could significantly increase the risk of SDE, while longer sleep duration was a protective factor for SDE. As reported, SDE participants might have poorer sleep quality, greater daytime sleepiness, more sleep disturbances, and higher incidence, prevalence, and severity of sleep disorders than non-SDE participants [58].

### Strengths and limitations

This study examined the effects of various types of RE on SDE, taking into account factors that might be associated with SDE from existing research, thereby updating the data on children’s ocular surface health. Additional benefits included a population-based design, a relatively large sample size and a high participation rate (90.4%).

However, our study had also encountered limitations. First, due to the limited time and venue, clinical data including ocular surface parameters (such as TUBT), ocular axis and cycloplegic-refraction were not measured, thus only focusing on the reported subjective symptoms through questionnaires and only exploring the association of different myopia degrees with SDE rather than refractive myopia and axial myopia. Second, the age of our participants was limited to 12–18 years, which could not represent children and teenagers of all ages. Third, since the limitations of cross-sectional studies, the causal relationship between risk factors and SDE cannot be clearly confirmed.

## Conclusion

This study revealed that high myopia was significantly associated with SDE among various types of RE. In addition, frequent use of eye drops, a history of allergic conjunctivitis, and frequent blinking were risk factors for SDE, while male gender, increased sleep time, and timely intervention when vision decline occurred were protective factors for SDE. Our findings will help provide valuable insights for future research and interventions aimed at improving ocular health in adolescents.

## Supporting information

S1 File (DOCX)
