# Supplementary material for: Prevalence of symptomatic dry eye and influencing factors among Chinese adolescents: A cross-sectional study
Source: PLoS One. 2024 Oct 29;19(10):e0312725. doi: 10.1371/journal.pone.0312725 (PMC11521286; doi:10.1371/journal.pone.0312725)
Supplement: S1 File — (DOCX) [file pone.0312725.s001.docx]

**Supplementary Material**

**Table-S1**: Self-designed questionnaire

| **Questionnaire** | | | | |
| --- | --- | --- | --- | --- |
| No. | subject | | | Answer |
| 1 | Age | Height (cm) | Weight (kg) |  |
| 2 | Sex: 1. Female; 2. Male | | |  |
| 3 | Use eye drops more than three times a week: 1. No; 2. Yes | | |  |
| 4 | Contact lens wear: 1. No; 2. Yes | | |  |
| 5 | Use of spectacles: 1. No; 2. Yes | | |  |
| 6 | Have good eating habits: 1. No; 2. Yes | | |  |
| 7 | Academic burden: 1. Normal; 2. Serious | | |  |
| 8 | Daily TV watching time: 1. ＜0.5 h; 2. 0.5-1 h; 3. 1-1.5 h; 4. 1.5-2 h; 5. ＞2 h | | |  |
| 9 | Daily mobile phone or iPad use time: 1. ＜0.5 h; 2. 0.5-1 h; 3. 1-1.5 h; 4. 1.5-2 h; 5. ＞2 h | | |  |
| 10 | Daily homework time: 1. ＜1 h; 2. 1-2 h; 3. 2-3 h; 4. 3-4 h; 5. ＞4 h | | |  |
| 11 | Daily sleep time: 1. ＜6 h; 2. 6-7 h; 3. 7-8 h; 4. ＞8 h | | |  |
| 12 | Daily outdoor activity: 1. ＜0.5 h; 2. 0.5-1 h; 3. 1-2 h; 4. 2-3 h; 5. ＞3 h | | |  |
| 13 | Timely intervention when vision loss occurred: 1. No; 2. Yes | | |  |
| 14 | History of allergic conjunctivitis: 1. No; 2. Yes | | |  |
| 15 | History of chalazion: 1. No; 2. Yes | | |  |
| 16 | Frequent blinking: 1. No; 2. Yes | | |  |
| 17 | Where mainly stay during class intervals: 1. Classroom; 2. Outdoor | | |  |
